# Targeting AXL Using the AVB-500 Soluble Receptor and through Genetic Knockdown Inhibits Bile Duct Cancer Growth and Metastasis

**DOI:** 10.3390/cancers15061882

**Published:** 2023-03-21

**Authors:** Jiyoung Kim, Gilyeong Nam, You Keun Shin, Nuria Vilaplana-Lopera, Hei-Cheul Jeung, Eui Jung Moon, Ik Jae Lee

**Affiliations:** 1 Department of Radiation Oncology, Gangnam Severance Hospital, Yonsei University College of Medicine, Seoul 06273, Republic of Korea; 2 MRC Oxford Institute for Radiation Oncology, Department of Oncology, University of Oxford, Headington OX3 7DQ, UK; 3 Department of Integrative Medicine, Gangnam Severance Hospital, Yonsei University College of Medicine, Seoul 06273, Republic of Korea; 4 Department of Oncology, Gangnam Severance Hospital, Yonsei University College of Medicine, Seoul 06273, Republic of Korea

**Keywords:** AXL, bile duct cancer, invasion, metastatsis, AVB-500

## Abstract

**Simple Summary:**

Bile duct cancer (cholangiocarcinoma) is a relatively rare cancer type that has a 5-year survival rate of 26% if diagnosed early, but a survival rate of less than 10% if the cancer has spread to the lymph nodes. Most patients with bile duct cancer are treated with surgery and chemotherapy, which unfortunately is more palliative than curative. In comparison to other gastrointestinal cancers, there are few targeted therapies which are specific to bile duct cancer. In searching for new molecular targets for bile duct cancer diagnosis and therapy, we found that the receptor tyrosine kinase AXL is highly expressed in bile duct cancer cells and that inhibition of its expression and signaling significantly reduces tumor growth and the spread of cancer cells. This study suggests that AXL is a potential new therapeutic target for treating bile duct cancers.

**Abstract:**

Bile duct cancer, or cholangiocarcinoma, is a rare disease with limited treatment options that include surgery and cytotoxic chemotherapy. The high recurrence rate and poor prognosis of this type of cancer highlights the need to identify new and more effective therapeutic targets. In this study, we found that AXL, a receptor tyrosine kinase, is highly expressed in biliary cancer patients and significantly correlated with poor patient outcomes, including metastasis and low survival rates. We also demonstrated that targeting AXL inhibits tumor progression. In vitro studies with bile duct cancer cells (SNU1196 and HUCCT1) showed that genetic knockdown of AXL significantly reduced both tumor cell growth and invasion. In addition, in vivo studies using subcutaneous and orthotopic intrahepatic models demonstrated that genetic inhibition of AXL resulted in tumor-growth delay. To further examine the possible clinical translation of AXL inhibition in the clinic, we tested the efficacy of AVB-500, a soluble AXL receptor, in reducing AXL activation and tumor growth. AVB-500 was effective at inhibiting AXL activation and decreasing the growth and invasion of SNU1196 and HUCCT1 tumors which possess high AXL expression. Most importantly, AVB-500 was highly effective at decreasing tumor dissemination of bile duct tumor cells in the peritoneal cavity. This study strongly supports the idea of using the AXL receptor as a new therapeutic target to treat the growth and progression of biliary cancer.

## 1. Introduction

Bile duct cancer, or cholangiocarcinoma, is a rare type of cancer that arises from the bile ducts which connect the liver, gallbladder and intestines. Based on the location, bile duct cancer can be classified as intrahepatic, perihilar or distal bile duct cancer [1]. While surgery remains the primary option for disease control, only a small number of patients are eligible for curative surgery, and the high relapse rate limits the 5-year survival to 5–15% [2]. In 2010, advanced biliary tract cancer (ABC)-02 phase III clinical trials demonstrated significant survival benefits from combination treatment with cisplatin and gemcitabine, which is now the standard first-line treatment for advanced biliary cancer [3]. Several treatments have been proposed as alternatives for patients who may not benefit from cisplatin and gemcitabine, including the combination of 5-fluorouracil, folinic acid and oxaliplatin (FOLFOX), which may have an advantage in disease control [4]. Recently, the use of pemigatinib, a small molecule targeting fibroblast growth factor receptor 2 (FGFR2), was approved for the treatment of advanced and metastatic cholangiocarcinoma patients in the US and the UK [5,6]. Additionally, another targeted therapy, ivosidanib, targeting isocitrate dehydrogenase-1 (IDH1) mutations, also showed promising results in phase III clinical trials with enhanced progression-free survival [7]. However, secondary mutations and acquired resistance to FGFR2 and IDH1 targeted therapies, along with the high toxicities observed in a phase II trial with cabozantinib, which targets VEGFR2, MET and AXL, indicate that it is crucial to identify new, specific targeted therapies that possess less normal tissue toxicity to treat bile duct cancer [8,9,10].

AXL is a receptor tyrosine kinase (RTK) and a member of the Tyro3-Axl-MERK (TAM) family [11,12]. When AXL binds to its ligand, GAS6, it dimerizes and activates multiple signaling pathways, including PI3K, MAPK and PKC. AXL is highly expressed in a variety of tumor types, including ovarian, breast, lung and renal cancers, and its expression correlates with poor patient survival through the regulation of invasion, metastasis, drug resistance and immune suppression [11,13,14,15,16,17,18,19]. The role of AXL in radiation responses and drug resistance makes it a potential therapeutic target for promoting anti-cancer treatment responses [13,20,21,22]. Currently, there are several small molecules that target AXL, including BGB324 (R428) and cabozantinib. However, these compounds are limited due to their non-specific effects on other RTKs, including Tie2, VEGFR, MET and Abl. The off target effects of small molecule inhibitors of AXL coupled with the high binding affinity of Gas6 for its receptor AXL led to the development of a soluble AXL decoy receptor with increased GAS6 binding affinity and specific AXL inhibition in a variety of cancers, including ovarian, renal, breast and pancreatic cancers [15,16,23]. Its efficacy in targeting AXL without causing significant normal tissue toxicity has been also validated in healthy volunteers and ovarian cancer patients [24,25].

This study examined whether AXL plays a significant role in the progression of bile duct cancers using both in vitro and in vivo systems. Additionally, we investigated the efficacy of AXL inhibition using both genetic knockdown and AVB-500, a soluble AXL decoy receptor, confirming AXL as a potential therapeutic target for bile duct cancer.

## 2. Materials and Methods

### 2.1. Patient Data Analysis

Expression levels of AXL and GAS6 mRNA in patient tissues of normal or cholangiocarcinoma were compared using The **U**niversity of **AL**abama at Birmingham **CAN**cer data analysis Portal (UALCAN), a web-based platform for differential gene expression (http://ualcan.path.uab.edu, last accessed 1 April 2022) [26,27]. Differential expression in N0 and N1 patients were also assessed. The raw data were obtained from the TCGA data portal (https://portal.gdc.cancer.gov/projects/TCGA-CHOL, last accessed 22 June 2022). An additional patient dataset from gene expression omnibus (GEO) was analyzed to determine the importance of AXL and GAS6 expression in cholangiocarcinoma patient tissues (GSE32225) with the inclusion of a proliferative subset of tissue samples.

### 2.2. Immunohistochemistry (IHC) and Analysis

Tissue microarray slides of patient tissues with normal and cholangiocarcinoma were purchased from US Biomax Inc, (#GA802A, #LV801b, Rockville, MD, USA), and IHC was performed as previously described [28]. Briefly, the slides were heated at 60 °C for 20 min, deparaffinized, and rehydrated using Citroclear (limonene-derivative, safer than xylene) × 2, 100% ethanol × 2, 70% ethanol, 50% ethanol, and dH_2_O for 3 min each. Then, the slides were immersed in 1 × citrate buffer diluted in dH_2_O with 0.05% Tween-20 (Sigma, #C9999, St. Louis, MO, USA) for 5 min at 110 °C and cooled to room temperature (RT) for 20 min. The endogenous enzymes were blocked using Dual Endogenous Enzyme Block (Agilent Technologies, #S2003, Santa Clara, CA, USA) at RT for 20 min, and the slides were washed with PBST (PBS with 0.05% Tween-20) and incubated with a human phospho-AXL (Y779) antibody (R&D Systems, Minneapolis, MN, USA, AF2228, 1:100) overnight at 4 °C. Slides were washed three times with PBST and incubated with HRP-labelled polymer anti-Rabbit (Agilent Technologies, # K4003) at RT for 20 min, and the signals were detected by *Diaminobenzidine* (DAB) chromogen/substrate (Agilent Dako, Santa Clara, CA, USA #K3468). The slides were rinsed in PBS, immersed in water for 5 min and counterstained with Gill’s Hematoxylin. After washing with water for 5 min, slides were mounted using Aquatex (Sigma #1085620050).

For hematoxylin and eosin (H&E) staining, after deparaffinization and rehydration, slides were immersed into Gill’s Hematoxylin for 10 s, washed with water for 5 min, and stained with eosin for 10 s. The slides were washed with water for 5 min, rehydrated, and mounted using Aquatex before imaging using an Aperio CS scanner and ImageScope analysis software v12.4.6.5003 (Aperio Technologies, Vista, CA, USA). DAB intensity and the liver tumor area were analyzed using ImageJ [29].

### 2.3. Cell Culture

KKU-M055, KKU-M213, MMNK1 and HUCCT1 cells were purchased from the Japanese Collection of Research Bioresources Cell Bank (JCRB, Osaka, Japan); and SNU245, SNU478, SNU869, SNU1079 and SNU1196 cells were purchased from the Korean Cell Line Bank (KCLB; Seoul, Republic of Korea). All cell lines were maintained in their specified medium supplemented with 10% FBS (SERANA) at 37 °C in a 95% air and 5% CO_2_ incubator. AXL shRNA in lentiviral was purchased and transfected into HEK293T cells (Sigma Aldrich #TRCN0000196945, 5′-GATTGCCATTGAGAGTCTAGC-3′). Cell media were collected 48 h after and filtered using a 0.45 µm syringe filter to collect viral particles. SNU1196 and HUCCT1 cells (2 × 10^5^ cells/well) were plated into a 96-well plate 24 h before incubation with media containing viral particles overnight at 37 °C. The media were removed and replaced with fresh culture media, and puromycin (2 mg/mL) was used to select stable AXL knockdown cells, which was confirmed by Western blotting. For GAS6 stimulation, cells were kept in serum-free medium for 24 h and then treated with 10 μg/mL or 100 μg/mL AVB-500 with the addition of 50 ng/mL GAS6 (R&D systems #885-GSB-050) for 24 h.

### 2.4. Growth Curve Analysis

Wild-type HUCCT1 and SNU1196 cells, and those infected with shScrambled control and shAXL, were seeded at a density of 5000 cells per well in a six-well plate. The cells were trypsinized after 24 h, and the number of cells was counted using an automated cell counter (CountessII, Thermo Fisher Scientific, Waltham, MA, USA). The cell counts were performed daily for 8 days.

### 2.5. Western Blot Analysis

Whole-cell lysates were extracted using CMRC lysis buffer containing 50 mM Tris-HCl (pH 7.4), 150 mM NaCl, 1% Triton X-100, 1 mM EDTA, 1 mM EGTA, 10% glycerol, 5 mM NaF and protease inhibitor cocktail. Protein was obtained by centrifugation at 12,000 rpm, 4 °C, for 15 min, and the concentration was determined by the BCA assay (Thermo Fisher Scientific). The protein samples were mixed with 4× protein sample buffer (Bio-Rad, Contra Costa County, CA, USA) and β-mercaptoethanol, and boiled at 95 °C for 5 min before electrophoresis in a 9.4% sodium dodecyl sulfate-polyacrylamide gel. The proteins were transferred to 0.45 µm polyvinylidene fluoride membranes (Millipore, Billerica, MA, USA), blocked with 5% skimmed milk in tris buffered saline/0.5% Tween-20 (TBST), and subsequently probed with primary antibodies against phospho-AXL (Tyr702) (1:1000, Cell signaling #5724S), AXL (1:2000, Santa Cruz #sc-1096), phospho-AKT (1:1000, Cell signaling #4060), AKT (1:1000, Cell signaling #9272), phospho-MEK (1:1000, Cell signaling #9121), MEK (1:1000, Cell signaling #9122) and β-actin (1:2000, Santa Cruz #sc-47778). After washing five times for 5 min with TBST, the membranes were incubated with secondary antibody for 2 h at room temperature and washed, and the protein bands were visualized with an ECL solution.

### 2.6. Trans-Well Invasion Assays

Matrigel invasion assays were performed according to the manufacturer’s protocol (Corning). Briefly, 1 × 10^6^ cells of HUCCT1, SNU 1196, SNU478 and SNU869 cells were seeded into the upper well of a Matrigel-coated Boyden chamber (8 μm pore size, Corning) in serum-free media. The bottom well contained 10% DMEM. After 24–48 h, the cells which had migrated through the Matrigel were fixed, stained and imaged in three different fields at 20× magnification. The experiments were performed in triplicate and repeated at least three times. Cell invasion was quantified by counting the number of invading cells per field. For AVB-500 treatment, the bottom chamber was filled with 700 μL culture media with 100 µg/mL AVB-500 for 24 h.

### 2.7. Wound Healing Assay

HUCCT1, SNU1196, SNU478 and SNU869 cells were plated into a 96-well plate at a density of 10,000 cells per well and allowed to grow in the culture medium until 95% confluency. The automated 96-pin wound-making tool WoundMakerTM (Essen Bioscience, Hertfordshire, UK) was then used to create a scratch wound, and images were taken automatically by the IncuCyte ZOOM^®^ (Essen Bioscience) every four hours up to 64 h. The IncuCyte wound-healing assay was conducted according to a predetermined procedure, and the relative wound density was calculated by IncuCyte™ Chemotaxis Cell Migration Software (Essen Bioscience). The data are presented as mean ± SD.

### 2.8. Cell Viability Assay

Cell viability was assessed using the Quanti-Max™ WST-8 Cell Viability Assay Kit (BIOMAX, Seoul, Republic of Korea) according to the manufacturer’s instructions. HUCCT1, SNU1196, SNU478 and SNU869 cells (1 × 10^4^ cells/100 μL/well) in 10% RPMI 1640 medium were plated in a 96-well plate and incubated for 24 h in the incubator at 37 °C with 5% CO_2_. Then, 100 μL of media with AVB-500 in a range of concentrations was added to each well. After 72 h, 10 μL of the Quanti-MAXTM mixture (BIOMAX, Seoul, Republic of Korea) was added to each well, and plates were incubated for 4 h before the absorbance was measured using a microplate reader at a wavelength of 450 nm.

### 2.9. Mouse Tumor Xenograft Model

Female athymic nude mice (7–8-weeks-old (nu/nu)) were purchased from Junbiotech and housed in pathogen-free laminar-flow cabinets. The Animal Care and Use Committee of Yonsei University’s College of Medicine approved the study protocols. For the subcutaneous tumor model, 5 × 10^6^ cells in 50 µL of PBS and 50 µL Matrigel were injected into the right flank of the nude mouse, and tumor growth was measured every day until the tumor size reached 1200 mm^3^ or for 30 days.

To establish the orthotopic bile duct cancer model, tumor cells (1 × 10^7^) were injected into the right liver area of nude mice. Briefly, each mouse was anesthetized with Alfaxalone, and the abdomens was sterilized with 70% ethanol before a 50 mm incision was vertically performed in the upper abdomen through the skin to expose the left lobe of the liver. Then, 1 × 10^7^ of SNU1196 cells were directly injected into the subcapsular parenchyma of the left lobe, and Surgicel (Ethicon, Somerville, NJ, USA) was applied to prevent bleeding and tumor cell spillage. The liver was gently placed back in the peritoneal cavity, and the abdominal wall and skin were sutured. Post-operative pain was treated with an analgesic. Thirty days after injection, the mice were sacrificed, and the liver tissues were excised and weighed to determine tumor growth.

The metastatic tumor model was established by injecting 5 × 10^6^ cells into the intraperitoneal cavity of nude mice. Five days after the injection, the metastatic tumor burden was confirmed based on the pilot study. Mice were randomized into three groups and injected with 50 mg/kg vehicle and 100 mg/kg of Soluble AXL targeting AXL pathways every two days for 25 days. Mice were euthanized by CO_2_ asphyxiation, followed by cervical dislocation, and the tumor mass in the intraperitoneal cavity was collected for IHC.

### 2.10. Statistical Analysis

Statistical analysis, including student’s *t*-tests, log-rank tests and one-way or two-way ANOVA with multiple comparisons, was performed using GraphPad. All error bars represent the mean ± SD.

## 3. Results

### 3.1. AXL Expression Correlates with Patient Survival in Bile Duct Cancer

**Figure 1:**
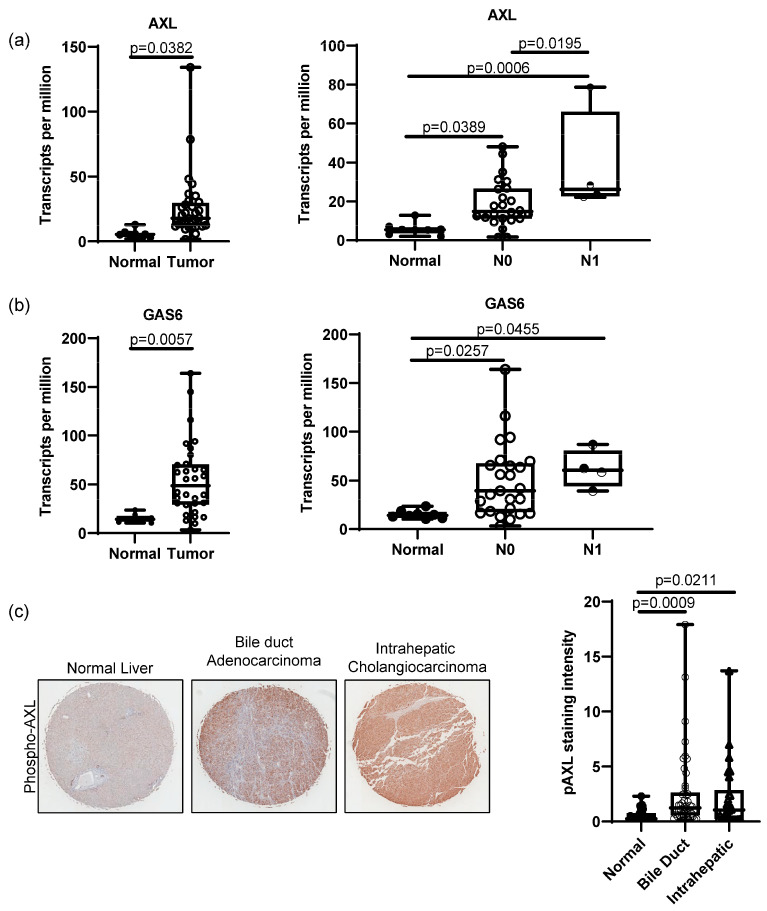
AXL expression in bile duct cancer patients. RNA sequencing data from TCGA analyzed by UALCAN showed that bile duct cancer tissues from patients highly expressed AXL (**a**) and GAS6 (**b**) compared to normal tissues (normal n = 8, bile duct cancer n = 32, *p =* 0.0382 for AXL and *p =* 0.0057 for GAS6, student’s *t*-test). UALCAN data present higher AXL expression in N1-stage (metastasis in 1–3 axillary lymph nodes) patients compared to normal and N0 (no metastasis) patients (normal n = 8, N0 n = 28, N1 n = 4; normal vs. N0 *p =* 0.0389, normal vs. N1 *p =* 0.0006, N0 vs. N1 *p =* 0.0195, one-way ANOVA with multiple comparisons). (**c**) Immunohistochemistry on TMA of cholangiocarcinoma (5 × magnification) patients exhibited significantly high phospho-AXL expression in cancer tissues compared to normal liver tissues. Staining intensity was analyzed using ImageJ (normal n = 45, bile duct cholangiocarcinoma n = 26, intrahepatic cholangiocarcinoma n = 46, normal vs. bile duct cholangiocarcinoma, *p =* 0.0009, normal vs. intrahepatic cholangiocarcinoma, *p =* 0.0211, one-way ANOVA with multiple comparisons).

To determine the role of AXL in bile duct cancer patients, we first evaluated mRNA expression of AXL in patient tissues with normal liver and cholangiocarcinoma using UALCAN, a web-based platform for differential gene expression based on publicly available omics data (TCGA, MET500, CPTAC and CBTTC) [26,27]. Data analysis from UALCAN showed that compared to normal tissues, tumor tissues from cholangiocarcinoma patients had significantly higher AXL expression (*p* = 0.0382, Figure 1a). AXL expression was also positively correlated with lymph-node metastasis, since patients with N1 cholangiocarcinoma (metastasis found in 1–3 axillary lymph nodes) had higher AXL expression than N0 patients (*p =* 0.0195, Figure 1a). Although GAS6 expression did not show a significant difference between N0 and N1 patients, it was still highly elevated in tumors when compared to normal tissues (*p =* 0.0057, Figure 1b). Activation of AXL in patients was further confirmed by IHC. A tissue microarray (TMA) of 75 patient tissue cores showed that phosphorylated AXL, a marker for AXL activation, is elevated in both bile ducts and intrahepatic cholangiocarcinoma tissues compared to 45 normal liver tissues (Figure 1c). Further analysis using the sGEO dataset (GSE32225) and cBioPortal also demonstrated elevated AXL expression in bile duct tumor tissues and suggested high AXL mRNA expression as a potential indicator of poor survival compared to those patients with low AXL expression, supporting the concept that dysregulated expression of AXL mRNA or activity is a prognostic marker for bile duct cancer (Supplementary Figure S1a,b). Although it did not meet statistical significance, expression of GAS6 still showed a similar trend of increased expression (Supplementary Figure S1a).

### 3.2. AXL Affects Tumor Cell Growth and Invasion/Migration in Bile Duct Cancer

**Figure 2:**
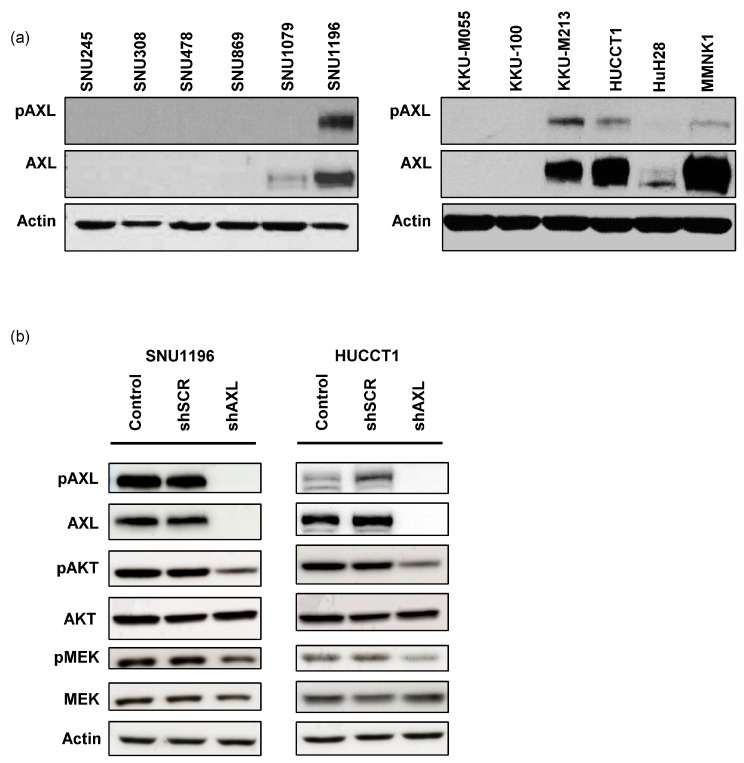
AXL expression in bile duct cancer cells. (**a**) The expression of AXL and phospho-AXL in bile duct cancer cell lines. In this study, we focused on SNU1196 and HUCCT1 with high expression of phospho-AXL and AXL. (**b**) In SNU1196 and HUCCT1 cells, AXL expression was knocked down using shRNA, resulting in reduced expression of phospho-AKT and phospho-MEK (shSCR: shRNA with scrambled sequences, shAXL: shRNA targeting AXL). Original blot see Supplementary File S1.

To investigate the functional role of AXL in bile duct cancer, AXL and phospho-AXL expression levels were evaluated in a variety of bile duct cancer cell lines (Figure 2a). Further analyses were performed on SNU1196 and HUCCT1, two cell lines overexpressing both AXL and phospho-AXL, whose growth in xenograft models has already been well characterized [30,31]. Genetic inhibition of AXL using shRNA confirmed reductions in AXL and phospho-AXL expression and in the downstream pathway activity, including AKT and MEK, in both cell lines (Figure 2b). The reduced activation of these pathways in bile duct cancer cells stably expressing shRNA against AXL suggests that activated AXL is a critical regulator of these pathways.

**Figure 3:**
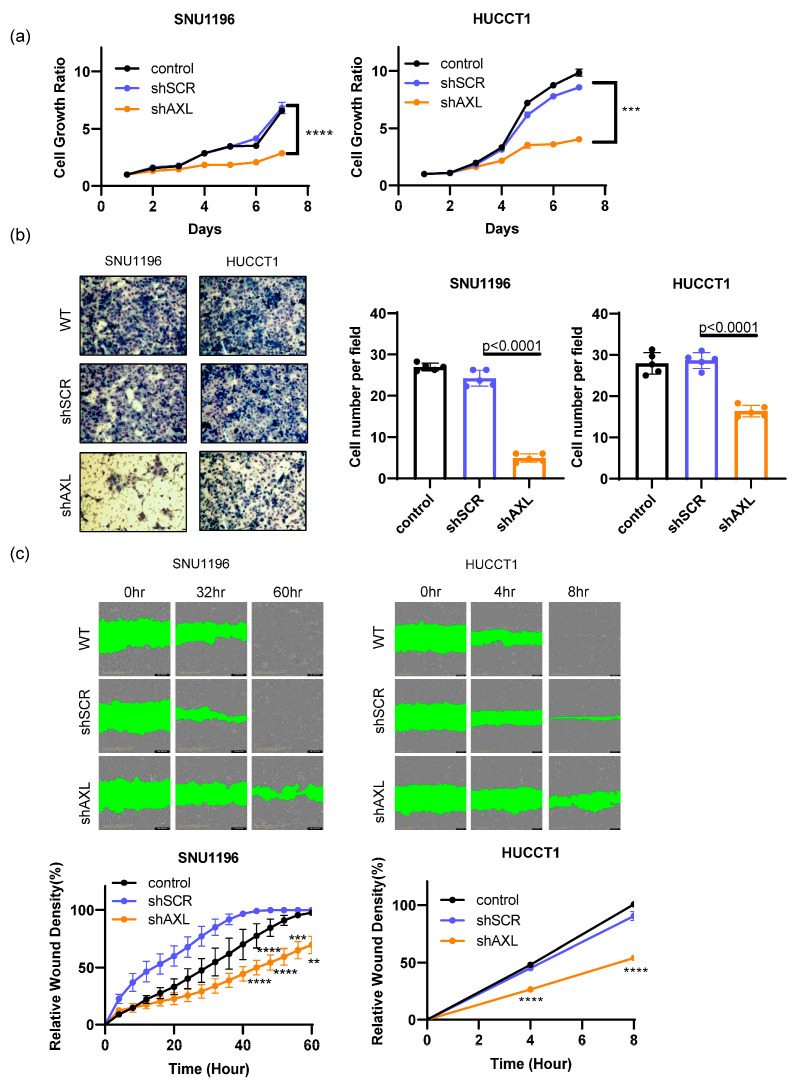
The roles of AXL in tumor cell growth and invasion/migration. (**a**) Inhibition of AXL expression using shRNA significantly decreased tumor cell growth in SNU1196 and HUCCT1 cells (two-way ANOVA, n = 3, *** *p* < 0.001, **** *p* < 0.0001). (**b**) Tumor cell invasion was assessed using Matrigel-coated invasion chambers. AXL inhibition significantly reduced the invasion of tumor cells through the Matrigel (two-way ANOVA, n = 5, *p* < 0.0001). (**c**) The role of AXL expression in tumor migration was determined by scratch assay. AXL knockdown significantly inhibited cell migration (two-way ANOVA, n = 3, ** *p* < 0.01, *** *p* < 0.001, **** *p* < 0.0001).

To determine whether AXL plays an essential role in the progression of bile duct cancer, we tested this hypothesis using the same AXL knockdown cells. Previous reports have suggested that AXL inhibition reduces tumor growth depending on cell types [15,16,18,23,32]. In our study, we found that genetic inhibition of AXL using shRNA resulted in reduced growth of both SNU1196 and HUCCT1 cells compared to control cells (Figure 3a). In addition to regulating tumor growth, AXL also plays a key role in tumor-cell invasion. Bile duct tumors with AXL shRNA exhibited impaired ability to invade through Matrigel (Figure 3b). The functional effect of AXL expression on cell migration was also evaluated using a scratch assay (Figure 3c). Although tumor growth rates were different in SNU1196 and HUCCT1 cell monolayers (64 h vs. 8 h), AXL inhibition induced the migration of both tumor cell lines to fill the wounded (scratched) area, demonstrating that AXL plays a critical role in regulating tumor cell migration. Therefore, these in vitro studies demonstrate that AXL is required for cell growth, invasion and migration in bile duct cancer cells.

### 3.3. AXL Regulates Tumor Growth In Vivo

**Figure 4:**
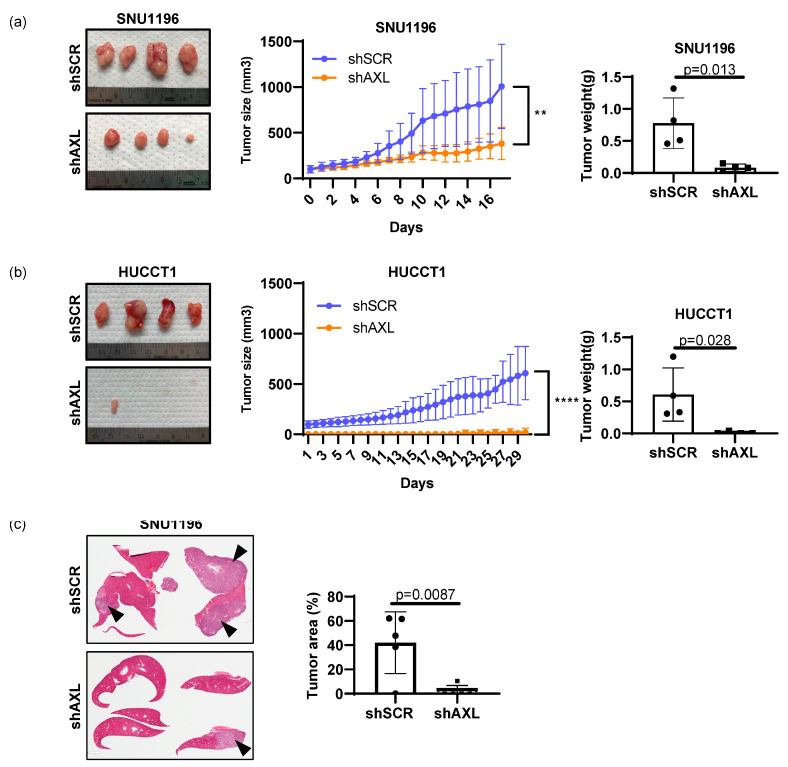
Regulation of tumor cell growth by AXL in vivo. The role of AXL in primary tumor growth was examined by subcutaneously growing (**a**) SNU1196 or (**b**) HUCCT1 in athymic nude mice. AXL knockdown significantly delayed tumor growth in both cell types (n = 4, two-way ANOVA, ** *p* < 0.01, **** *p* < 0.0001 for HUCCT1). Tumor weight measurement confirmed tumor growth delay (n = 4, student’s *t*-test, *p =* 0.013 for SNU1196 and *p =* 0.028 for HUCCT1). (**c**) The orthotopic model of bile duct cancer determined that AXL expression is essential for the intrahepatic growth of SNU1196 cells, since intrahepatic tumor growth was significantly inhibited in the absence of AXL expression (n = 5 for each group of shScrambled and shAXL, student’s *t*-test *p =* 0.0087, arrows indicate tumor areas).

To further investigate whether genetic inhibition of AXL affects tumor growth in vivo, SNU1196 or HUCCT1 cells were subcutaneously injected into the right flanks of immunodeficient nude mice, and tumor growth was measured daily. Unlike the in vitro data, HUCCT1 tumors grew slower than SNU1196 in mice (Figure 4a,b). More importantly, AXL knockdown significantly reduced subcutaneous tumor growth of both cell lines in mice. Tumor weight was measured at the end point after collecting tumor tissues from mice. Differences in tumor weight between shScrambled and shAXL tumor tissues further support that inhibition of AXL expression decreased tumor growth.

Since both SNU1196 and HUCCT1 are derived from intrahepatic cancers, SNU1196 cells were injected directly into the liver to recapitulate the microenvironment of bile duct tumors (Figure 4c). Histological analysis of liver tissues demonstrates that inhibition of AXL expression by genetic knockdown also decreases tumor growth in the orthotopic setting as well.

### 3.4. Soluble AXL Decoy Receptor Inhibits AXL-Mediated Tumor Growth and Invasion/Migration

**Figure 5:**
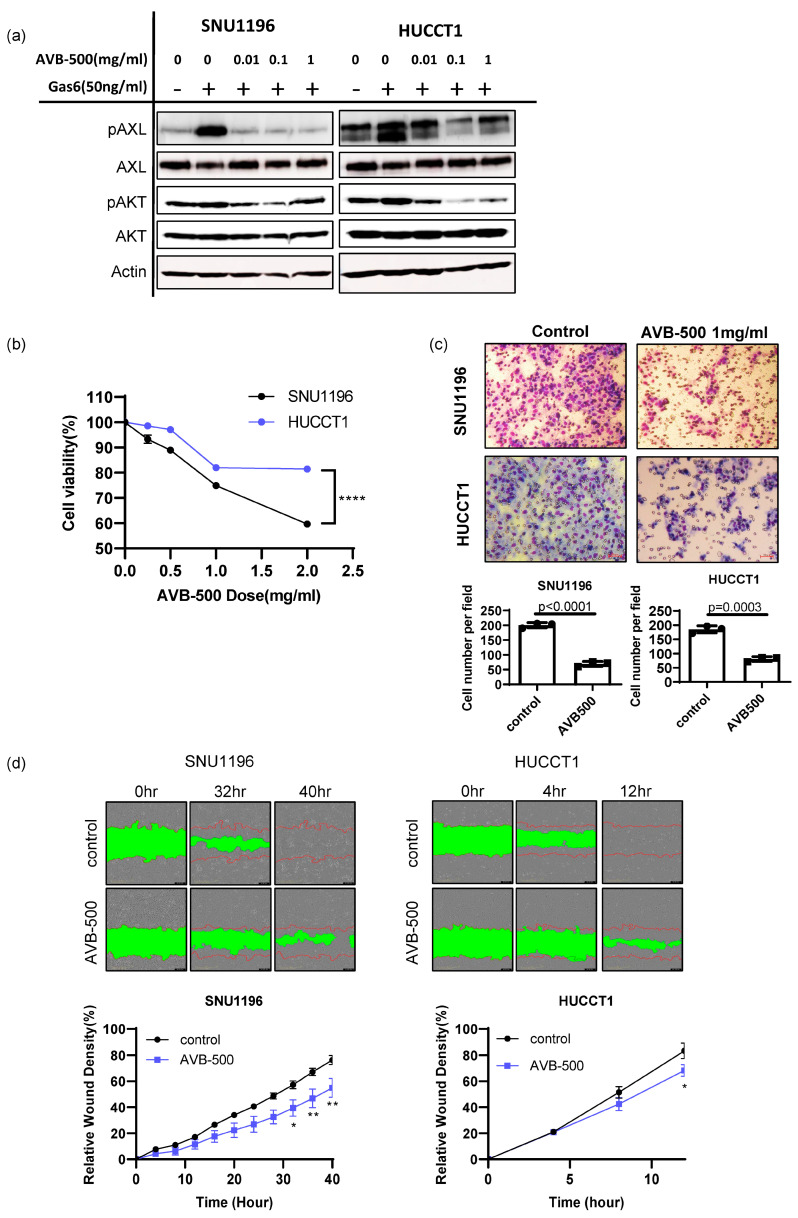
Evaluation of AVB-500, a soluble AXL that is able to decrease tumor growth and invasion in vitro. (**a**) When cells were stimulated with GAS6 after starvation, AVB–500 treatment (0.01, 0.1, 1 mg/mL) decreased phospho–AXL and phospho–AKT expression. (**b**) AVB–500 treatment dose-dependently decreased tumor–cell survival (n = 6, two–way ANOVA with multiple comparisons **** *p* < 0.0001). (**c**) Inhibition of AXL by 1 mg/mL of AVB–500 significantly reduced tumor–cell invasion in both SNU1196 and HUCCT1 cells (n = 3, student’s *t*–test *p* < 0.0001 for SNU1196, *p =* 0.0003 for HUCCT1)(200 × magnification). (**d**) Scratch assays showed that AXL inhibition by AVB-500 significantly inhibited cell migration (two-way ANOVA, n = 3, * *p* < 0.05, ** *p* < 0.01).

AVB-500 (formerly AVB-S6-500) is a recombinant fusion protein containing an extracellular region of human AXL fused with a human immunoglobulin G1 (IgG1) heavy chain (Fc). With high binding affinity to GAS6, its potential efficacy in inhibit tumor growth, invasion and metastasis has been extensively studied in ovarian, pancreatic, breast and renal cancer cancers [15,16,23]. More importantly, a recent phase Ib clinical trial in platinum-resistant ovarian cancer patients demonstrated its safety and clinical efficacy [25]. Therefore, we investigated AVB-500 as a potential therapeutic agent in bile duct cancer. Consistently with the genetic knockdown study, soluble AXL treatment reduced AXL activation (phospho-AXL and phospho-AKT) and tumor-cell survival in a dose-dependent manner (Figure 5a,b).

The effect of soluble AXL in tumor-cell invasion was also examined using an invasion chamber assay. We found that the number of tumor cells invading the Matrigel was reduced when SNU1196 and HUCCT1 cells were treated with 1 mg/mL of AVB-500 (Figure 5c). The scratch assay further supported that inhibition of AXL activation using AVB-500 decreased the migratory potential of SNU1196 and HUCCT1 (Figure 5d). In contrast, AVB-500 treatment did not induce any cytotoxicity in SNU478 and SNU869 biliary cancer cells, which do not express AXL (Supplementary Figure S2a). In addition, it did not alter tumor invasion, indicating that AVB-500’s inhibitory effect is specific to AXL (Supplementary Figure S2b).

**Figure 6:**
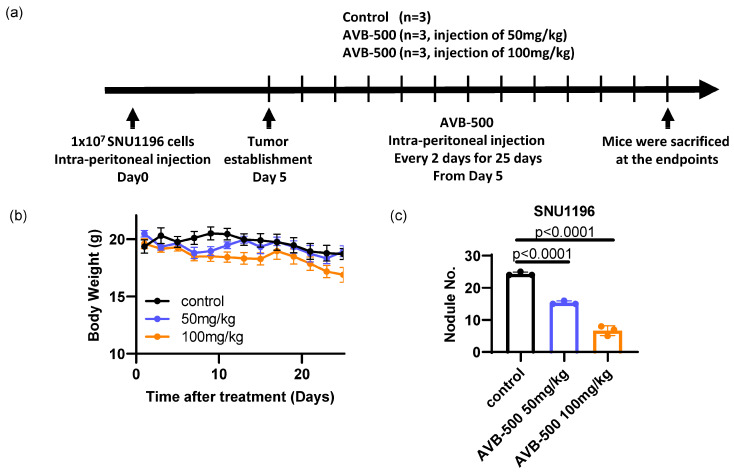
Evaluation of AVB–500, a soluble AXL that is able to decrease tumor growth and invasion in vivo. (**a**) A metastatic mouse model of bile duct cancer was established by injecting SNU1196 into the peritoneal cavity of an athymic nude mouse. While the mice did not show overall body weight changes (**b**), the number of tumor nodules was significantly decreased by AVB–500 treatment (**c**) (n = 3 per each group, one–way ANOVA with multiple comparisons, *p* < 0.0001 (vehicle vs. 50 mg/kg AVB–500, vehicle vs. 100 mg/kg)).

Although surgery is the most curative treatment for bile duct cancer patients, at the time of diagnosis, 50% of patients typically exhibit lymph-node metastasis and 10–20% metastasis in the peritoneal sites [33]. Thus, metastatic lesions were established through intraperitoneal injection of SNU1196 in immunodeficient nude mice (Figure 6a). While AVB-500 treatment (50 mg/kg or 100 mg/kg) did not cause changes in overall body weight for mice (Figure 6b), the number of tumor nodules in the intraperitoneal cavity was significantly reduced, indicating that a soluble AXL receptor can decrease the metastatic tumor burden in bile duct cancer without inducing side effects (Figure 6c).

## 4. Discussion

Bile duct cancer is a rare form of cancer which exhibits high relapse rates and poor prognosis due to limited treatment options. Clearly, there is a crucial clinical need to identify new therapeutic targets. This study—making use of TCGA and GEO data for differential gene expression, on the basis of AXL mRNA expression levels found in normal compared to cholangiocarcinoma patients—identified AXL as an essential factor regulating tumor progression in bile duct cancer-affected patients with metastasis and poor prognosis [26,27]. The analysis of AXL-expressing bile duct cancer cells (SNU1196, HUCCT1) revealed that AXL plays significant roles in tumor growth, invasion and metastasis. Although there are mixed results regarding the action of AXL in primary tumor growth, our data showed that knocking down AXL inhibited cell growth in vitro and tumor growth in vivo. Our studies indicate that AXL might be a good therapeutic target for controlling intrahepatic bile duct cancer at its primary site [15,16,17,18,23,32]. The role of AXL in tumor-cell invasion and migration was also confirmed using Matrigel-coated invasion chambers and scratch assays.

A recent phase II trial in bile duct cancer with cabozantinib, a multi-kinase inhibitor targeting VEGFR2, MET and AXL, supports targeting receptor tyrosine kinases such as AXL as possible therapeutic targets to treat cholangiocarcinoma [10]. However, the study showed that the high toxicity of cabozantinib limits its use in cholangiocarcinoma patients. In addition, the use of other targeted treatments, including pemigatinib (FGFR2) and ivosidanib (IDH1), is hindered by mutation-mediated drug resistance [8,9]. While there is a strong need to identify more potent and specific therapeutic targets, our data support AVB-500 to control both primary and metastatic bile duct cancers. The anti-tumor effect of AVB-500 has been confirmed in ovarian, breast, pancreatic and renal cancers [15,16,23], where it inhibits tumor growth and metastasis while simultaneously sensitizing tumors to chemotherapeutic treatment. The low toxicity of soluble AXL was confirmed in cynomolgus monkeys by using up to 150 mg/kg dose levels [24]. Its safety and efficacy were also determined in healthy volunteers and ovarian cancer patients [24,25]. The present study showed that AVB-500 treatment reduces tumor growth and invasion in AXL-expressing bile duct cancer cells (SNU1196, HUCCT1) compared to non-AXL expressing tumor cells (SNU478 and SNU869), suggesting that AXL expression and activation can be used as diagnostic factors to identify patient groups who will most likely benefit from AXL-targeted therapy. Moreover, the in vivo metastasis model in the intraperitoneal cavity confirmed that soluble AXL treatment effectively reduced metastatic burden. Overall, the present study demonstrates AXL inhibition as a potential therapy to inhibit both bile duct tumor growth and metastasis.

## 5. Conclusions

Our study demonstrated that AXL expression is highly upregulated in cholangiocarcinoma patients and potentially can be used as a prognostic marker. Inhibition of AXL using both shRNA and a soluble AXL, AVB-500, showed significant decreases in tumor growth and metastasis. Therefore, AXL is a potential therapeutic target for the treatment of bile duct cancer.

## Data Availability

Datasets used for bioinformatic analysis are available in cbioportal (cbioportal.org) for cholangiocarcinoma, TCGA, PanCancer Atlas and gene expression omnibus (GEO, GSE32225, ncbi.nlm.nih.gov/geo).

## Supplementary Materials

The following supporting information can be downloaded at: https://www.mdpi.com/article/10.3390/cancers15061882/s1, Figure S1: AXL expression in cholangiocarcinoma patients; Figure S2: The effect of AVB–500 in non-AXL expressing cells. Flie S1: Original blot.
