# Supplementary material for: Targeting AXL Using the AVB-500 Soluble Receptor and through Genetic Knockdown Inhibits Bile Duct Cancer Growth and Metastasis
Source: Cancers (Basel). Author manuscript; Available in PMC 2023 Apr 18. (PMC10047303; doi:10.3390/cancers15061882)

Supplementary Figure S1

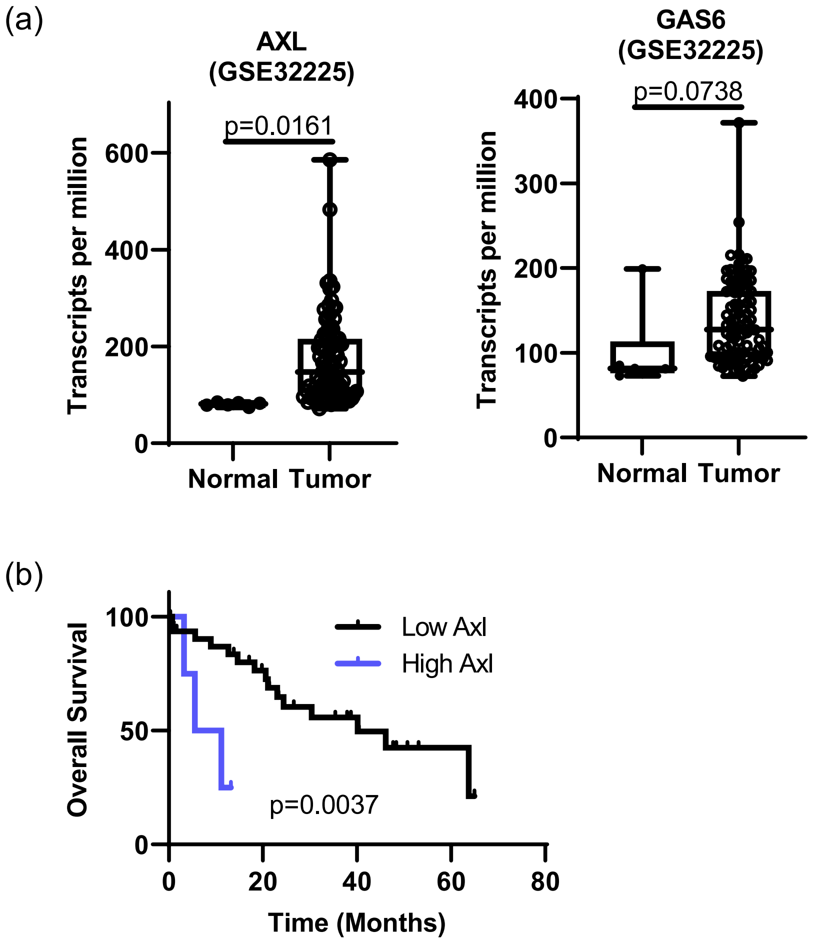

Supplementary Figure S1. AXL expression in cholangiocarcinoma patients. (a) Analysis of AXL and GAS6 expression in publicly available GEO dataset (GSE32225) showed that AXL is significantly upregulated in cholangiocarcinoma tissues in comparison to normal liver (Normal n = 6, Tumor n = 92, p = 0.0161, student's t-test). Although it did not meet statistical significance, GAS6 expression showed a trend of increases in tumor tissues when it was compared with normal liver (Normal n = 6, Tumor n = 92, p = 0.0738, student's t-test). (b) Data from the cBioPortal determined high expression of AXL significantly correlated with poor patient survival in cholangiocarcinoma (low AXL expression n = 32, high AXL expression n = 4, p = 0.0037, log-rank test).

Supplementary Figure S2

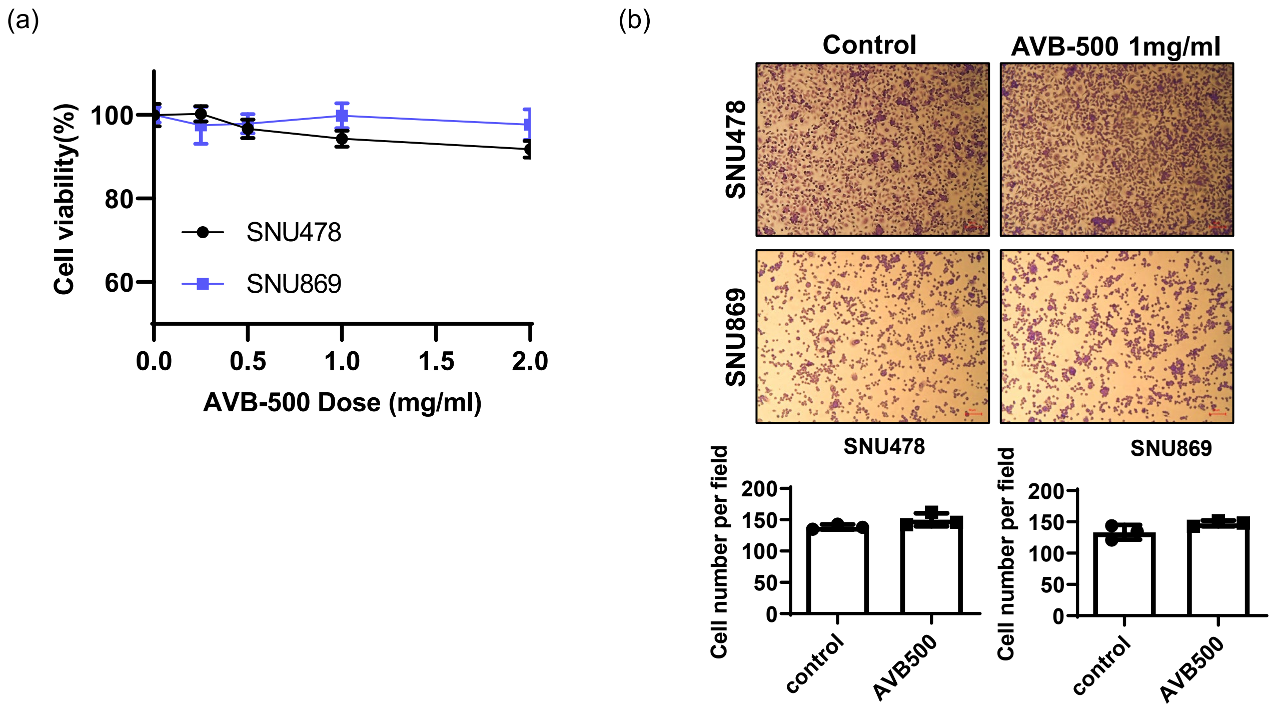

Supplementary Figure S2. The effect of AVB–500 in non-AXL expressing cells. (a) In bile duct cancer cells with no AXL expression (SNU478 and SNU869), treatment of AVB–500 did not induce cell cytotoxicity (n = 6). (b) In SNU478 and SNU869, 1mg/ml of AVB–500 did not inhibit tumor cell invasion (n = 3).

Supplementary Files S1. Raw Western Blot

Figure 2 (a)

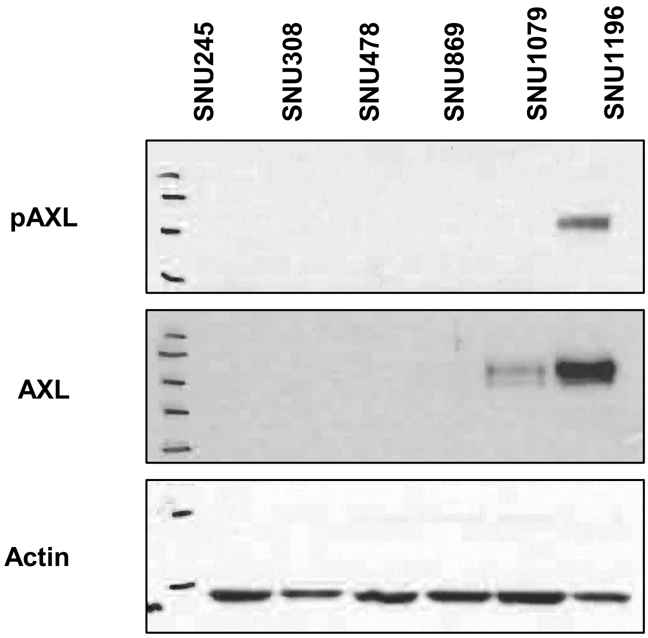

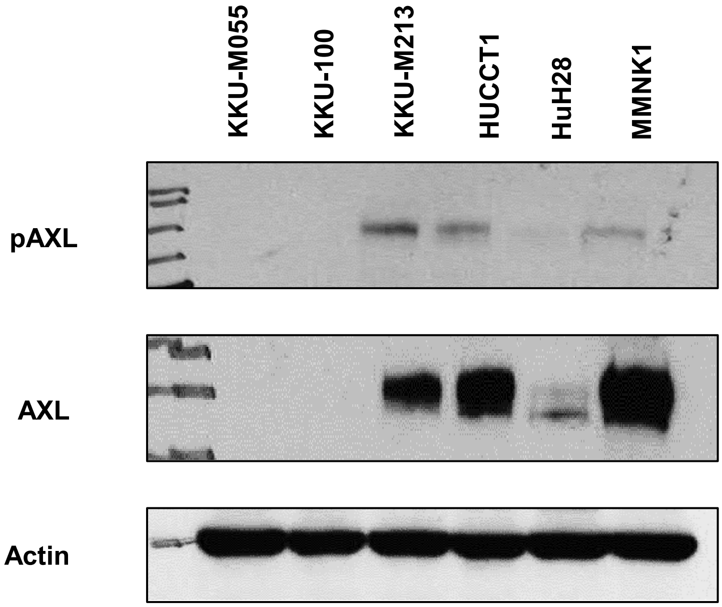

Supplementary Files S1. Raw Western Blot

Figure 2(b)

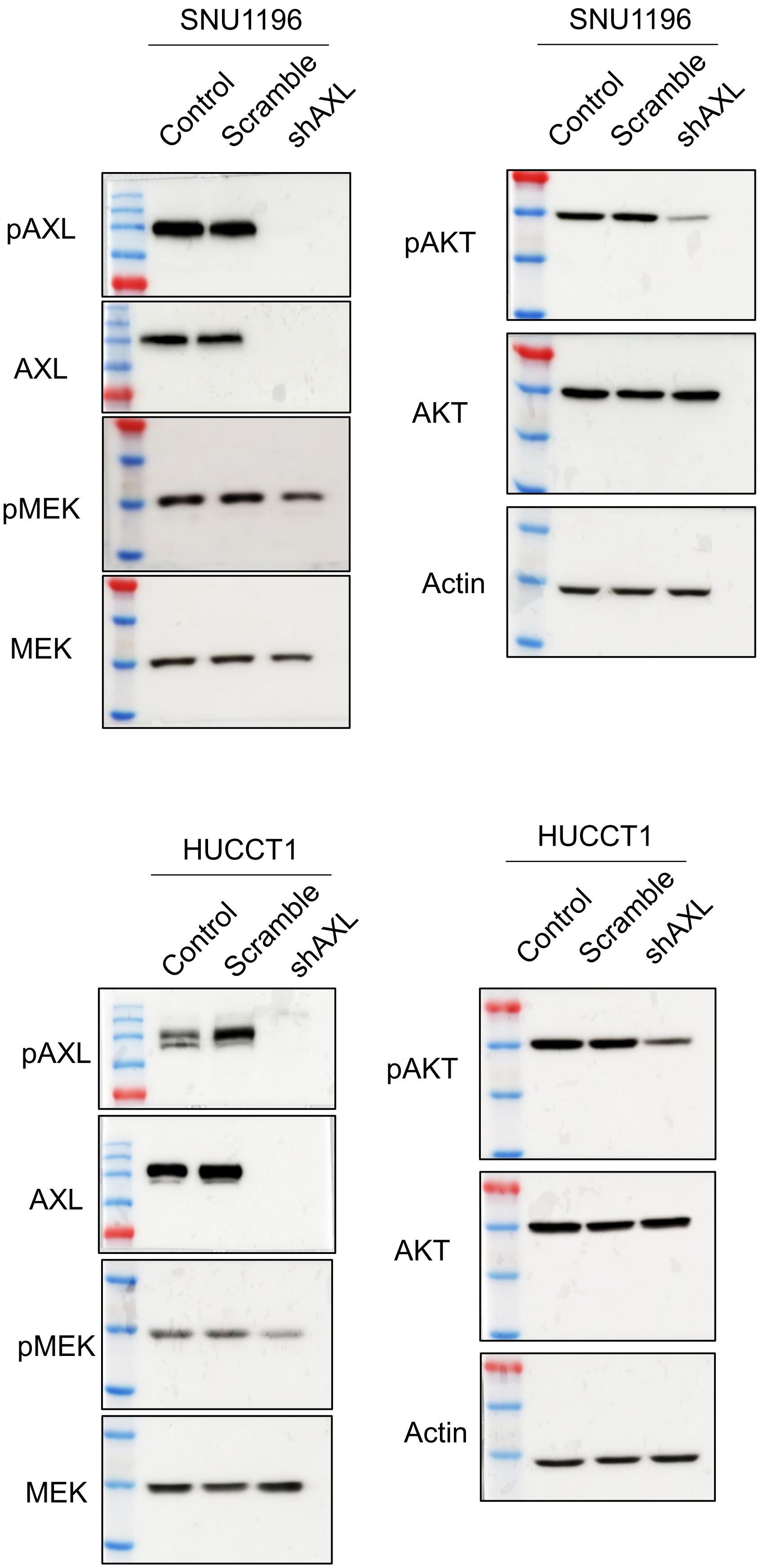

Supplementary Files S1. Raw Western Blot

Figure 5 (a)

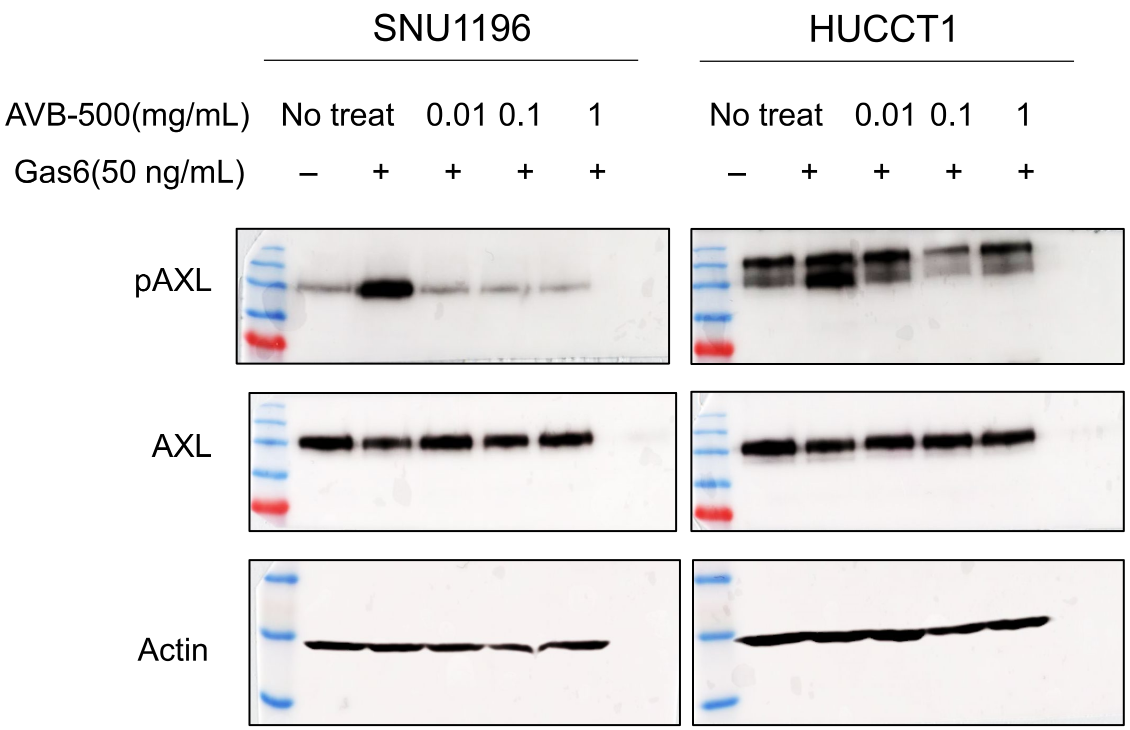

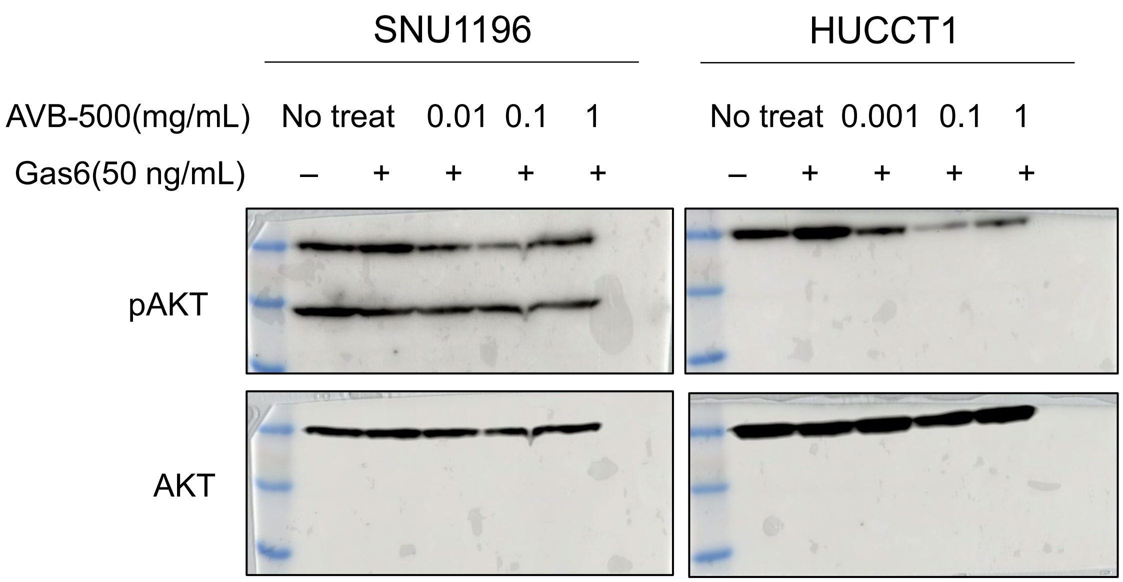

Supplement: Supplementary files [file EMS172746-supplement-Supplementary_files.pdf]
